# The safety of cyclosporine and tacrolimus in pediatric nephrotic syndrome patients: a disproportionate analysis based on the FAERS database

**DOI:** 10.3389/fped.2024.1487441

**Published:** 2025-01-07

**Authors:** Yu Liu, Chong Yan, Yaowang Zhao, Sui Deng, Jiancheng Zu

**Affiliations:** ^1^ Department of Urology, The Affiliated Children’s Hospital of Xiangya School of Medicine, Central South University (Hunan Children’s Hospital), Changsha, China; ^2^ Changde Hospital, Xiangya School of Medicine, Central South University (The First People’s Hospital of Changde City), Changde, China

**Keywords:** tacrolimus, nephrotic syndrome, pediatrics, FAERS, real-world data, cyclosporine

## Abstract

**Objective:**

This study aimed to systematically evaluate the safety of cyclosporine (CsA) and tacrolimus (TAC) in pediatric nephrotic syndrome (NS) patients using real-world data from the FDA Adverse Event Reporting System (FAERS).

**Methods:**

We analyzed adverse event (AE) reports from the FAERS database between Q4 2003 and Q2 2024, focusing on AEs associated with CsA and TAC in NS patients aged 18 years and younger. We employed three signal detection methods—Proportional Reporting Ratio (PRR), Relative Reporting Ratio (RRR), and Reporting Odds Ratio (ROR)—to assess the risk of drug-related AEs. Sensitivity analyses were conducted to explore the influence of gender on AE occurrence.

**Results:**

A total of 207 CsA-related and 145 TAC-related AE reports were included. CsA was significantly associated with nephropathy toxic (ROR = 8.26, 95% CI: 4.21–16.20), urine output decreased (ROR = 29.93, 95% CI: 3.66–244.61), and posterior reversible encephalopathy syndrome (ROR = 6.70, 95% CI: 3.17–14.14). TAC was associated with an increased risk of dystonia (ROR = 67.93, 95% CI: 8.63–534.86), kidney fibrosis (ROR = 22.65, 95% CI: 8.16–62.87), and diabetic ketoacidosis (ROR = 46.51, 95% CI: 5.68–380.97). Sensitivity analysis indicated that gender influenced the occurrence of AEs, with CsA showing higher nephrotoxicity in male patients, while TAC was more strongly associated with metabolic disorders and neurological AEs in female patients.

**Conclusion:**

In pediatric NS patients, CsA primarily induces nephrotoxicity and neurological complications, whereas TAC is more likely to cause kidney fibrosis and metabolic disorders. Enhanced monitoring of these AEs and individualized drug adjustments based on patient characteristics are recommended to optimize treatment outcomes and reduce AE incidence.

## 1 Introduction

Nephrotic syndrome (NS) is a common chronic kidney disease in childhood, with an incidence of approximately 2–7 cases per 100,000 children annually. The characteristic features of NS include massive proteinuria, hypoalbuminemia, hyperlipidemia, and generalized edema, which severely affect the patients' quality of life and long-term prognosis (1). About 10% of NS patients are resistant to glucocorticoid therapy, developing steroid-resistant nephrotic syndrome (SRNS), which typically requires treatment with immunosuppressants (2). Cyclosporine (CsA) and tacrolimus (TAC) are the primary immunosuppressants used in the treatment of SRNS.

CsA, available under brand names such as *Neoral®* and *Sandimmune®*, is typically administered orally or intravenously in doses ranging from 2 to 5 mg/kg/day, divided into two doses. The formulations include capsules, oral solutions, and injectable solutions. The duration of CsA therapy in SRNS patients can range from months to several years, depending on the patient's response and tolerance. CsA has been widely used in the treatment of SRNS since the 1980s. Studies have shown that approximately 50%–70% of pediatric SRNS patients treated with CsA achieve complete or partial remission within the first 6 months of therapy (3). Meta-analyses have also demonstrated that CsA significantly increases the number of children with SRNS achieving complete remission compared to placebo or no treatment (4). However, CsA use is associated with significant nephrotoxicity, including elevated serum creatinine levels and tubulointerstitial fibrosis, which may lead to irreversible kidney damage (5). Additionally, CsA is closely associated with adverse effects such as hypertension, gingival hyperplasia, and hirsutism, which significantly impact the patient's quality of life (6).

In contrast, TAC, a newer calcineurin inhibitor, has been increasingly used in SRNS treatment due to its stronger immunosuppressive effects and lower metabolic side effects compared to CsA. TAC, marketed under brand names like *Prograf®* and *Astagraf XL®*, is primarily administered orally, with starting doses of 0.1–0.2 mg/kg/day in two divided doses. Formulations include capsules and extended-release tablets. TAC is generally favored for its stronger immunosuppressive effect and reduced risk of hirsutism and gingival hyperplasia compared to CsA. In the short term, TAC has been shown to be more effective than CsA in treating pediatric SRNS (7). Other studies have shown that 6 months of TAC therapy results in complete or partial remission in 95% of patients, with a high remission rate and lower nephrotoxicity observed even after one year of follow-up (8).

Currently, there is a lack of comprehensive safety evaluations of CsA and TAC in pediatric NS patients. It is crucial to evaluate the safety of these drugs specifically in pediatric populations, as children may respond differently to these medications due to their developing organ systems and physiological characteristics. For instance, children's kidneys are still maturing, which affects the metabolism and clearance of drugs like CsA and TAC, potentially leading to altered side effect profiles. With the widespread use of real-world data (RWD), large-scale RWD-based assessments of drug safety have become an important trend in pharmacovigilance research (9). The FDA Adverse Event Reporting System (FAERS), a large-scale database based on voluntary reporting, provides rich real-world data that offers valuable resources for evaluating drug-related AEs in different populations (10). The database has significant advantages in discovering rare adverse reactions. Disproportionality analysis, a standard method in pharmacovigilance, provides insights into potential associations between drugs and AEs by comparing observed vs. expected AE frequencies. This study aims to analyze the AE characteristics of CsA and TAC in pediatric NS patients using the OpenVigil tool, based on FAERS data, and systematically evaluate their safety in this specific population. We hypothesized that CsA and TAC may have different risks of adverse reactions in children with nephrotic syndrome. This will provide clinicians with the latest evidence on the safety of CsA and TAC, guiding more rational drug use strategies.

## 2 Materials and methods

### 2.1 Data source and collection

The FAERS database was accessed through the OpenVigil 2.1 platform (https://openvigil.sourceforge.net/), which provides cleaned and standardized FAERS data. This study included reports submitted between Q4 2003 and Q2 2024. The FAERS database was chosen for its extensive coverage of global drug safety data, its availability for public research, and its acceptance as a reliable source for pharmacovigilance studies. OpenVigil 2.1 cleans and standardizes FAERS data, providing a large-scale adverse event database that is accessible for researchers to query and analyze (11). This study focused specifically on CsA and TAC, restricting the analysis to cases where these drugs were identified as the Primary Suspect (PS) and including only reports related to nephrotic syndrome patients aged 18 years and younger. Adverse events were coded using the Medical Dictionary for Regulatory Activities (MedDRA), version 25.1. Drugs were classified according to the Anatomical Therapeutic Chemical (ATC) classification system. Reports with ambiguous drug assignments (e.g., concomitant use without clear attribution to primary or secondary suspect drugs) were excluded to avoid bias.

### 2.2 Statistical analysis

The primary signal detection in this study was conducted using three disproportionality analysis methods: Proportional Reporting Ratio (PRR) (12), Relative Reporting Ratio (RRR) (13), and Reporting Odds Ratio (ROR) (14). Disproportionality analysis is a standard methodology in pharmacovigilance to detect signals of drug-related AEs by comparing the observed vs. expected reporting frequencies of specific AEs. These methods evaluate whether a specific drug is disproportionately associated with certain AEs compared to other drugs in the database. A 2 × 2 contingency table was used to calculate these measures, as shown Table 1. These methods were employed to assess the significance of adverse events associated with CsA and TAC in pediatric nephrotic syndrome patients compared to other drugs. Additionally, to address potential sources of bias, sensitivity analyses were conducted. First, reports were stratified by gender to explore potential differences in AE profiles between male and female patients.TAC. Second, to assess the robustness of the findings, the analysis was repeated without restricting the drug's role to the primary suspect. All statistical analyses were performed using R software (version 4.1.2).

**Table 1:** 2 × 2 contingency table for disproportionality analysis.

	Drug of interest (e.g., CsA, TAC)	All other drugs
AE of interest	a	b
All other AEs	c	d

The disproportionality measures are calculated as follows: PRR=(a/(a+c))/(b/(b+d));ROR=(a/c)/(b/d);RRR=(a/(a+b))/(c/(c+d)).

## 3 Results

### 3.1 Basic characteristics of adverse event reports

We retrieved a total of 1,063 adverse event reports related to pediatric nephrotic syndrome patients, of which 207 were primarily associated with CsA and 145 were primarily associated with TACCsATAC, as detailed in Table 2. In terms of gender distribution, 61.8% of the CsA group were male and 35.3% were female, while in the TAC group, 50.3% were male and 44.8% were female. Regarding age distribution, both CsA and TAC reports predominantly involved children aged 6–12 years, accounting for 37.2% and 35.2% of cases, respectively. The geographical distribution of the reports showed that the majority of CsA-related adverse events were reported from Japan (57.0%), whereas TAC-related reports were primarily from the United States (26.9%), Canada (12.4%), and China (11.0%).

**Table 2:** Basic characteristics of adverse event reports related to CsA and TAC.

Characteristics	CsA	TAC
All AEs	207	145
Gender		
Males	128 (61.8)	73 (50.3)
Females	73 (35.3)	65 (44.8)
Not reported	6 (2.9)	7 (4.8)
Age groups		
0–2	35 (16.9)	17 (11.7)
3–5	51 (24.6)	45 (31.0)
6–12	77 (37.2)	51 (35.2)
13–18	44 (21.3)	32 (22.1)
Not reported	0 (0.0)	0 (0.0)
Reporter country		
Canada	7 (3.4)	18 (12.4)
China	4 (1.9)	16 (11.0)
France	6 (2.9)	15 (10.3)
India	4 (1.9)	12 (8.3)
Italy	6 (2.9)	6 (4.1)
Japan	118 (57.0)	7 (4.8)
United States	19 (9.2)	39 (26.9)
Germany	4 (1.9)	1 (0.7)
United Kingdom	3 (1.4)	5 (3.4)
Poland	6 (2.9)	4 (2.8)
Other countries	26 (12.6)	6 (4.1)
Not reported	4 (1.9)	16 (11.0)
Reporter year		
2004–2008	35 (16.9)	2 (1.4)
2009–2013	46 (22.2)	35 (24.1)
2014–2018	60 (29.0)	34 (23.4)
2019–2023	60 (29.0)	70 (48.3)
2024	6 (2.9)	4 (2.8)

### 3.2 Risk signal detection results

**Figure 1:**
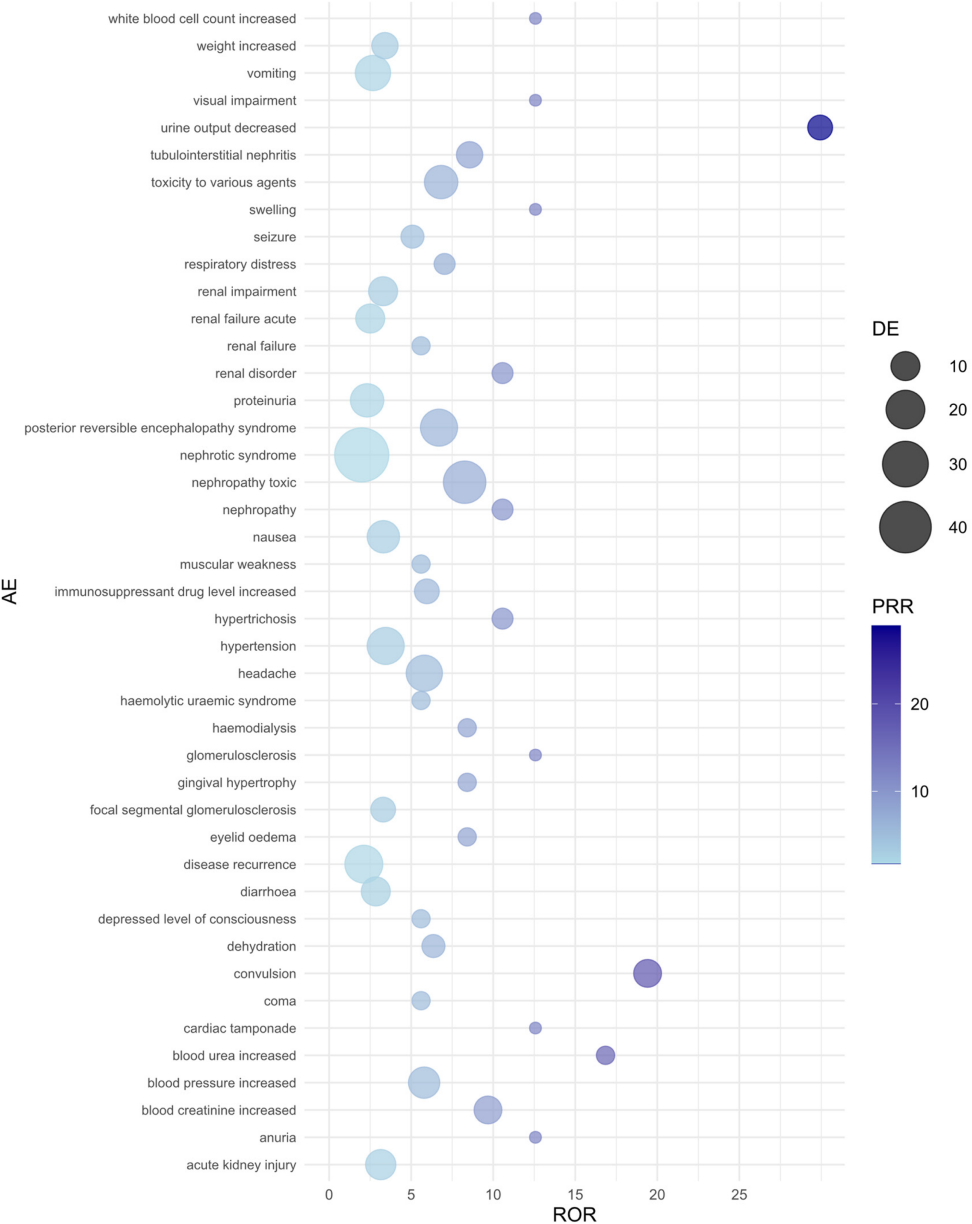
Bubble plot of adverse events associated with cyclosporine use. AE, adverse event; ROR, reporting odds ratio; PRR, proportional reporting ratio; DE, represents the number of reports for a specific adverse event suspected to be primarily related to the drug of interest. For example, a DE of 9 means that cyclosporine has been reported as primarily suspected in 9 cases of a specific adverse event.

**Figure 2:**
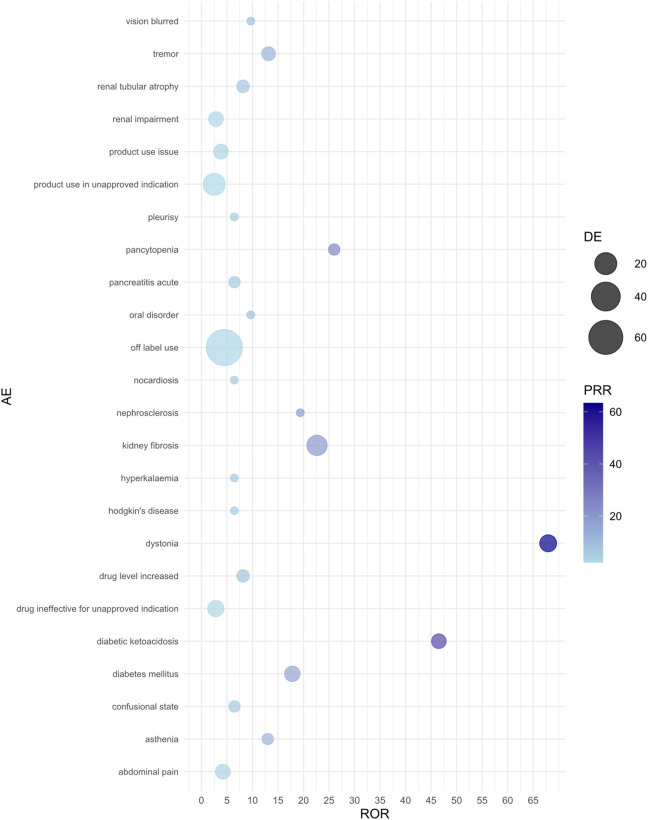
Bubble plot of adverse events associated with tacrolimus use. AE, adverse event; ROR, reporting odds ratio; PRR, proportional reporting ratio; DE, represents the number of reports for a specific adverse event suspected to be primarily related to the drug of interest. For example, a DE of 9 means that tacrolimus has been reported as primarily suspected in 9 cases of a specific adverse event.

The background population used for disproportionality analysis comprised pediatric nephrotic syndrome patients (aged ≤18 years) within the FAERS database, including reports for all medications. Reports that did not involve CsA or TAC served as the reference group. This approach allowed for a comparison of adverse events associated with CsA and TAC against those reported for a broader range of drugs in the FAERS database, ensuring robust signal detection. In pediatric nephrotic syndrome patients, we analyzed the risk of each adverse event associated with CsA or TAC, identified as the primary suspect drug, using the ROR, PRR, and RRR methods. Tables 3, 4 present the top 20 adverse events for each drug, ranked by the lower bound of the ROR 95% confidence interval (CI), with all three signal detection methods indicating a significant risk. Figures 1, 2 show the risk mining results for AEs associated with the primary suspicion of CsA and TAC, respectively.

**Table 3:** Safety of CsA in pediatric nephrotic syndrome patients.

Adverse event	PRR	Chi_squared	RRR	ROR (95% CI)
Nephropathy toxic	7.38	48.51	3.29	8.26 (4.21–16.20)
Convulsion	18.61	23.68	4.20	19.41 (4.16–90.53)
Urine output decreased	28.95	19.62	4.49	29.93 (3.66–244.61)
Posterior reversible encephalopathy syndrome	6.20	29.73	3.08	6.70 (3.17–14.14)
Blood creatinine increased	9.30	17.69	3.56	9.68 (2.95–31.76)
Toxicity to various agents	6.43	23.06	3.13	6.83 (2.91–16.00)
headache	5.41	24.85	2.91	5.80 (2.77–12.15)
Tubulointerstitial nephritis	8.27	14.33	3.42	8.56 (2.55–28.72)
Blood pressure increased	5.51	17.01	2.93	5.79 (2.41–13.94)
Hypertrichosis	10.34	9.02	3.67	10.57 (2.04–54.87)
Nephropathy	10.34	9.02	3.67	10.57 (2.04–54.87)
Renal disorder	10.34	9.02	3.67	10.57 (2.04–54.87)
Blood urea increased	16.54	8.18	4.11	16.85 (1.87–151.54)
Immunosuppressant drug level increased	5.79	9.32	3.00	5.96 (1.87–18.96)
Hypertension	3.24	14.65	2.26	3.45 (1.83–6.52)
Dehydration	6.20	8.13	3.08	6.36 (1.78–22.74)
Respiratory distress	6.89	6.95	3.21	7.04 (1.67–29.69)
Nausea	3.16	9.70	2.23	3.31 (1.58–6.92)
Seizure	4.96	6.61	2.80	5.08 (1.54–16.81)
Eyelid oedema	8.27	5.81	3.42	8.41 (1.53–46.26)

**Table 4:** Safety of TAC in pediatric nephrotic syndrome patients.

Adverse event	PRR	Chi_squared	RRR	ROR (95% CI)
Dystonia	63.31	49.90	6.67	67.93 (8.63–534.86)
Kidney fibrosis	20.26	65.84	5.59	22.65 (8.16–62.87)
Diabetic ketoacidosis	44.32	31.28	6.42	46.51 (5.68–380.97)
Diabetes mellitus	16.88	28.07	5.33	17.81 (4.67–67.95)
Tremor	12.66	17.36	4.89	13.17 (3.26–53.25)
Off label use	2.81	69.89	2.25	4.49 (3.11–6.49)
Pancytopenia	25.32	13.55	5.87	26.01 (2.89–234.43)
Asthenia	12.66	10.23	4.89	12.99 (2.36–71.60)
Drug level increased	7.91	10.19	4.07	8.16 (2.17–30.76)
Renal tubular atrophy	7.91	10.19	4.07	8.16 (2.17–30.76)
Nephrosclerosis	18.99	8.14	5.50	19.37 (2.00–187.54)
Confusional state	6.33	6.20	3.67	6.48 (1.60–26.21)
Pancreatitis acute	6.33	6.20	3.67	6.48 (1.60–26.21)
Oral disorder	9.50	5.64	4.40	9.68 (1.60–58.42)
Vision blurred	9.50	5.64	4.40	9.68 (1.60–58.42)
Abdominal pain	4.03	7.85	2.85	4.18 (1.59–10.97)
Product use issue	3.69	6.95	2.70	3.83 (1.48–9.90)
Product use in unapproved Indication	2.30	10.46	1.96	2.51 (1.46–4.33)
Hodgkin's disease	6.33	4.02	3.67	6.44 (1.29–32.24)
Hyperkalaemia	6.33	4.02	3.67	6.44 (1.29–32.24)

For CsA-related adverse events, nephropathy toxic (ROR = 8.26, 95% CI: 4.21–16.20) and urine output decreased (ROR = 29.93, 95% CI: 3.66–244.61) showed the strongest associations. Additionally, CsA use significantly increased the risk of convulsion (ROR = 19.41, 95% CI: 4.16–90.53) and blood creatinine increased (ROR = 9.68, 95% CI: 2.95–31.76). Other high-risk adverse events included posterior reversible encephalopathy syndrome, headache, tubulointerstitial nephritis, and blood pressure increased, all of which exhibited high ROR values. The PRR and RRR analyses also supported the association between CsA and these adverse reactions.

In adverse event reports related to TAC, dystonia (ROR = 67.93, 95% CI: 8.63–534.86) and kidney fibrosis (ROR = 22.65, 95% CI: 8.16–62.87) showed the strongest associations. Additionally, diabetic ketoacidosis (ROR = 46.51, 95% CI: 5.68–380.97) and diabetes mellitus (ROR = 17.81, 95% CI: 4.67–67.95) also significantly increased the risk during TAC use. Other high-risk adverse events include tremor, pancytopenia, increased drug level, and renal tubular atrophy, all of which exhibited high ROR values. The results from the PRR and RRR methods similarly supported the association between TAC and these adverse reactions.

### 3.3 Reported outcomes

**Figure 3:**
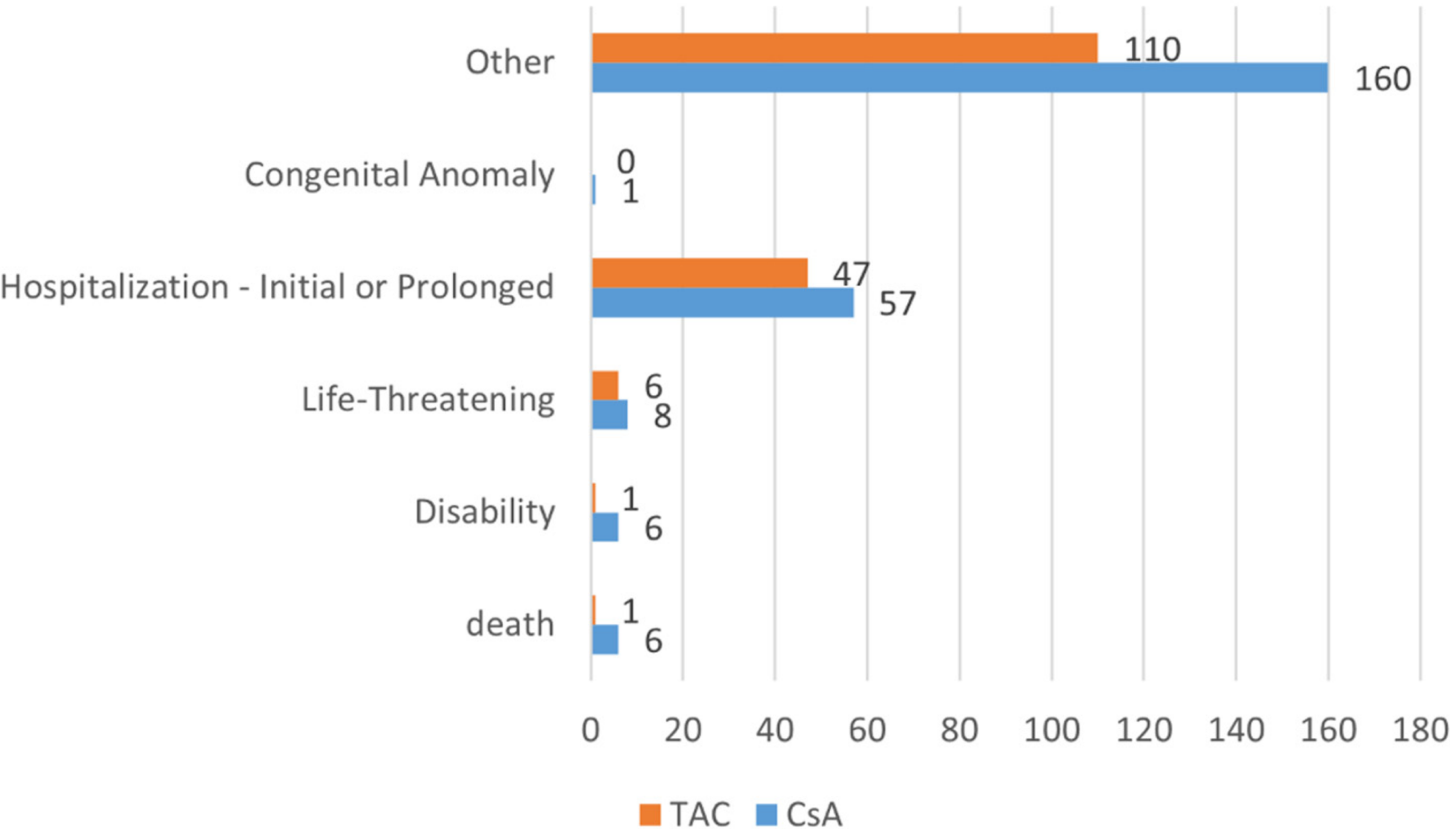
Adverse event outcomes in pediatric nephrotic syndrome patients treated with cyclosporine and tacrolimus. CsA, cyclosporine; TAC, tacrolimus.

The adverse event outcomes listed in Table 5 were included based on their clinical relevance and actual reporting frequency in the FAERS database. These outcomes reflect the severity of the reported adverse events and provide important insights for clinicians and regulatory agencies. In pediatric nephrotic syndrome patients, the reported outcomes of adverse reactions associated with CsA and TAC predominantly involved “other serious outcomes” and “hospitalization” (including both initial and prolonged hospitalizations due to adverse reactions). As shown in Figure 3, the CsA group reported 6 cases ofdeath and disability respectively, while the TAC group reported 1 case of these outcomes. Life-threatening adverse events were reported in 8 cases in the CsA group and 6 cases in the TAC group. Additionally, the CsA group reported 1 case of congenital anomaly.

**Table 5:** Common adverse reactions associated with CsA and TAC use.

CsA		TAC	
Adverse event	ROR (95% CI)	Adverse event	ROR (95% CI)
Nephropathy toxic	7.62 (3.33–17.43)	Off label use	4.12 (3.01–5.64)
Hypogammaglobulinaemia	4.95 (2.39–10.24)	Kidney fibrosis	10.25 (3.72–28.25)
Hypertension	2.27 (1.20–4.28)	Drug ineffective for unapproved indication	2.38 (1.14–4.97)
Proteinuria	2.42 (1.27–4.60)	Pneumocystis jirovecii pneumonia	5.39 (2.10–13.83)
Posterior reversible encephalopathy syndrome	4.46 (1.97–10.12)	Therapy non-responder	2.44 (1.09–5.44)
Agranulocytosis	2.10 (1.08–4.07)	Product use issue	3.46 (1.39–8.60)
Headache	3.77 (1.71–8.31)	Weight increased	3.10 (1.22–7.89)
Renal failure acute	4.62 (1.94–11.02)	Cough	6.21 (1.86–20.80)
Toxicity to various agents	3.66 (1.49–8.97)	Diabetic ketoacidosis	21.74 (2.66–177.50)
Kidney fibrosis	4.00 (1.54–10.39)	Covid-19	4.62 (1.29–16.51)

### 3.4 Sensitivity analysis

**Figure 4:**
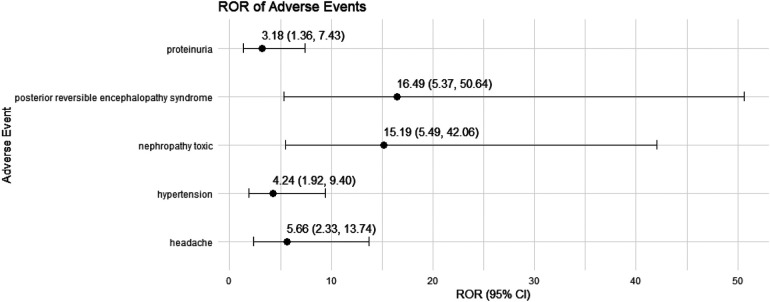
Risk signals for cyclosporine in male pediatric nephrotic syndrome patients. ROR, reporting odds ratio.

**Figure 5:**
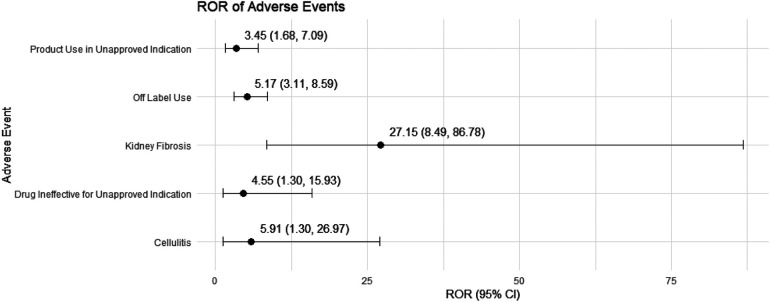
Risk signals for tacrolimus in male pediatric nephrotic syndrome patients. ROR, reporting odds ratio.

We conducted a sensitivity analysis across different genders in pediatric nephrotic syndrome patients. In male patients, CsA remained highly associated with nephropathy toxic (ROR = 15.19, 95% CI: 5.49–42.06) and posterior reversible encephalopathy syndrome (ROR = 16.49, 95% CI: 5.37–50.64). Additionally, there were significant associations with hypertension, headache, and proteinuria, as shown in Figure 4. TAC continued to show a strong risk signal for kidney fibrosis (ROR = 27.15, 95% CI: 8.49–86.78). Furthermore, in male pediatric nephrotic syndrome patients, TAC was associated with an increased risk of cellulitis (ROR = 5.91, 95% CI: 1.30–26.97). Risks related to inappropriate drug use were also observed, including drug ineffective for unapproved indication (ROR = 4.55, 95% CI: 1.30–15.93) and off-label use (ROR = 5.17, 95% CI: 3.11–8.59), as illustrated in Figure 5.

**Figure 6:**
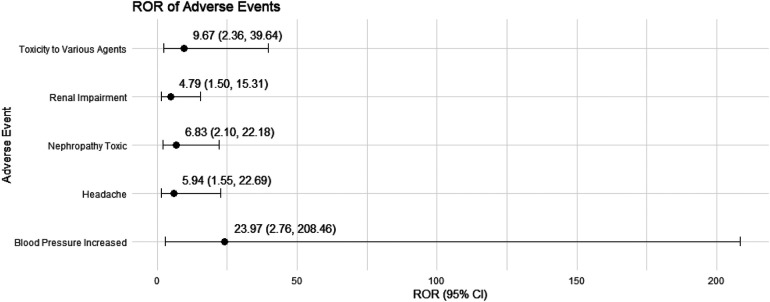
Risk signals for cyclosporine in female pediatric nephrotic syndrome patients. ROR, reporting odds ratio.

**Figure 7:**
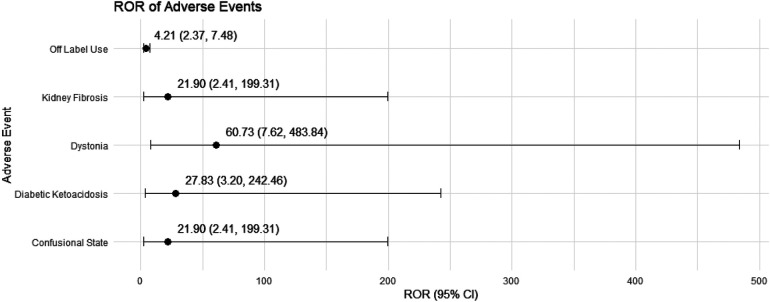
Risk signals for tacrolimus in female pediatric nephrotic syndrome patients. ROR, reporting odds ratio.

In female pediatric nephrotic syndrome patients, CsA exhibited the strongest risk signal for blood pressure increased (ROR = 23.97, 95% CI: 2.76–208.46), followed by toxicity to various agents (ROR = 9.67, 95% CI: 2.36–39.64). Additional significant associations were observed with nephropathy toxic (ROR = 6.83, 95% CI: 2.10–22.18), renal impairment (ROR = 4.79, 95% CI: 1.50–15.31), and headache (ROR = 5.94, 95% CI: 1.55–22.69), as presented in Figure 6. TAC was strongly associated with dystonia (ROR = 60.73, 95% CI: 7.62–483.84) and diabetic ketoacidosis (ROR = 27.83, 95% CI: 3.20–242.46). Moreover, TAC showed significant risks for kidney fibrosis (ROR = 21.90, 95% CI: 2.41–199.31) and confusional state (ROR = 21.90, 95% CI: 2.41–199.31). There was also a potential for inappropriate use, such as off-label use (ROR = 4.21, 95% CI: 2.37–7.48), as depicted in Figure 7.

In a further analysis, we removed the restriction on the role of the drug as the primary suspect to explore the common adverse reactions associated with CsA and TAC use. The top 10 adverse reactions by frequency, all of which showed significant risk signals across the three signal detection methods, are presented in Table 5. CsA was primarily associated with nephropathy toxic, acute renal failure (reported in FAERS, clinically known as acute kidney injury), posterior reversible encephalopathy syndrome, hypertension, and headache, all of which displayed strong risk signals. TAC showed a strong association with diabetic ketoacidosis and kidney fibrosis, with significant risks also observed in cases of clinical misuse.

### 3.5 External validity

The external validity of these results is supported by several factors. First, the FAERS database includes reports from multiple countries, reflecting diverse clinical practices and patient populations. Second, the key findings, such as nephrotoxicity associated with CsA and metabolic disorders linked to TAC, are consistent with existing literature, suggesting reproducibility. Third, by leveraging spontaneous reports, this study captures real-world drug use patterns and safety profiles, offering insights that controlled trials may not fully address. Nevertheless, the inherent limitations of spontaneous reporting systems, such as reporting bias and underreporting, warrant caution in interpreting these findings.

## 4 Discussion

This study systematically evaluated the safety of CsA and TAC in pediatric nephrotic syndrome patients through an in-depth analysis of the FAERS database. Our findings indicate that CsA use is significantly associated with nephrotoxicity, particularly manifested as reduced urine output, elevated serum creatinine levels, and a high incidence of acute renal failure (reported in FAERS, clinically known as acute kidney injury). Previous studies have demonstrated that CsA induces nephrotoxicity by inhibiting calcineurin, leading to intracellular calcium homeostasis disruption, oxidative stress, and inflammatory responses in renal tubular epithelial cells (5). This prolonged cellular stress promotes the development of tubulointerstitial fibrosis, ultimately resulting in irreversible renal damage (15). Moreover, inhibition of cyclophilin chaperone function with ensuing endoplasmic reticulum (ER) stress and proapoptotic unfolded protein response (UPR) aggravates CsA toxicity, whereas pharmacological modulation of UPR holds potential to mitigate renal side effects of CsA (16). CsA may also exacerbate renal ischemia and damage by inducing vasoconstriction and reducing renal blood flow (17). Research indicates that CsA-induced hypertension is associated with sympathetic nervous system activation, which is more pronounced in heart transplant patients (18). Omega-3 fatty acids (3 g/day) can lower blood pressure by reducing systemic vascular resistance (19), and nitroglycerin has been shown to counteract CsA-induced high systolic blood pressure to some extent (20). A study by Louhelainen M et al. found that CsA-induced myocardial ANP and connective tissue growth factor (CTGF) mRNA overexpression, RAS activation, NADPH oxidase induction, and NRF2 overexpression were prevented by the natural antioxidant lipoic acid (LA). LA also induced the mRNA expression of gamma-glutamylcysteine ligase, the rate-limiting enzyme in glutathione synthesis, and significantly increased hepatic cysteine and glutathione concentrations (21).

We also found a significant association between CsA and posterior reversible encephalopathy syndrome (PRES). Previous studies have reported similar associations (22–24). Danish A et al. reported that in allogeneic stem cell transplant recipients, the incidence of CsA-induced neurotoxicity (CIN) could reach up to 13% (25). The mechanism of PRES may involve CsA-induced hypertension and disruption of cerebrovascular autoregulation (26). Additional studies have suggested that the main factor associated with CsA neurotoxicity is systemic hypertension, though other factors such as CsA-induced vascular changes or hypoalbuminemia may also play a role, with intracranial hemorrhage potentially occurring due to related thrombocytopenia (27). Moreover, the clinical and imaging manifestations of CsA neurotoxicity appear to be similar to those of hypertensive encephalopathy (24). Regression analysis has shown that age <6 years (OR 7.6, 95% CI 1.6–51.5; *p* = 0.007) and post-CsA hypertension (OR 6.3, 95% CI 1.4–35.4; *p* = 0.016) are significantly associated with CsA encephalopathy (28). Younger children are prone to more severe seizures during the acute phase, and are at higher risk of developing epilepsy and neuropsychiatric disorders in the future. Therefore, when using CsA, it is crucial to pay special attention to blood pressure management and early identification of neurological symptoms in pediatric patients to prevent severe neurological complications.

Compared to CsA, TAC exhibits different characteristics in terms of nephrotoxicity. Our study shows a significant increase in the risk of kidney fibrosis associated with TAC, potentially through a mechanism where TAC induces fibroblast-to-myofibroblast transition via a TGF-β-dependent pathway, contributing to renal fibrosis (29). Liu L et al. proved that TAC upregulates NORAD to compete with miR-136-5p, resulting in a decrease in miR-136-5p expression, which in turn activates the TGF-β1/smad3 pathway, ultimately leading to the aggravation of renal fibrosis (30). Although TAC is more effective in the short term for treating SRNS compared to CsA (7), long-term use may accelerate renal fibrosis, leading to the progression of chronic kidney disease (31). Furthermore, our study found a strong association between TAC and diabetic ketoacidosis, likely due to TAC' toxic effects on pancreatic β-cells and inhibition of insulin secretion (32–34). Studies have shown that both TAC and CsA inhibit calcineurin and nuclear factor of activated T cells (NFAT) function under high glucose and palmitate conditions, indicating that these drugs may induce β-cell dysfunction through shared pathways, with TAC having a more pronounced effect on β-cell function compared to CsA (35). This metabolic disorder risk is particularly significant in pediatric patients, as their endocrine systems are not yet fully developed, potentially resulting in lower tolerance to the drug. Other studies have shown that conversion from CsA to TAC in renal transplant patients led to an increase in fasting blood glucose levels from 101.7 ± 18.5 to 107.4 ± 21.3 mg/dl (*P* = .007) and an increase in glycated hemoglobin levels from 5.7 ± .8 to 6.0 ± 1.2% (*P* = .016) (36).

TAC also demonstrated a strong association with dystonia; however, this potential adverse reaction has not been widely reported. In contrast, CsA has been previously reported to cause dystonia (37, 38). Therefore, this adverse reaction may be underrecognized in clinical practice, but its severity warrants enhanced neurological monitoring during TAC therapy. Additionally, our study revealed a degree of inappropriate use of TAC in clinical practice, such as off-label use and use in unapproved indications. Therefore, it is recommended to strengthen education and management regarding TAC use in clinical practice to ensure its safe and effective application.

Sensitivity analysis results showed CsA tends to exhibit higher nephrotoxicity in males, which may be related to higher levels of oxidative stress and the effects of testosterone, factors that enhance the damage to renal tubules. TAC is more commonly associated with metabolic and neurological adverse events in females, possibly due to the influence of estrogen, gender differences in metabolic pathways, and immune system variations. For example, testosterone may enhance the nephrotoxicity of CsA because it may promote oxidative stress (39) and tubular damage (40). In contrast, estrogen has a certain protective effect on the metabolism of TAC, but it may also affect the metabolic pathways.

Despite the important clinical insights provided by this study, several limitations should be acknowledged. First, the voluntary reporting mechanism of the FAERS database may lead to reporting bias, and the lack of detailed information on dosage, the comorbidities and concomitant drugs and treatment duration could affect the accuracy of the results. Variations in reporting rates across countries may reflect differences in access to healthcare, diagnostic practices, regulatory frameworks, and pharmacovigilance systems. For instance, stricter post-marketing surveillance in some regions might lead to higher reporting rates, while limited access to healthcare in others could contribute to underreporting. Additionally, changes in prescribing patterns, such as the increasing preference for TAC over CsA due to its lower nephrotoxicity, may influence the observed safety profiles. These factors introduce potential bias and should be carefully considered when interpreting the results. Moreover, signals of disproportionate reporting, as detected in this study, do not establish causation and cannot be used to calculate incidence rates or make direct risk comparisons between drugs. Lastly, as we used secondary data, we did not account for a sufficient number of covariates, and did not conduct a more comprehensive sensitivity analysis. Additionally, we were unable to explore the dose-response relationship in depth. Therefore, these results should be considered as hypothesis-generating and require further validation through complementary research methodologies. Future research should incorporate prospective cohort studies and randomized controlled trials to more comprehensively assess the safety of CsA and TAC in pediatric nephrotic syndrome patients, incorporating demographic factors such as age groups, ethnicity, and comorbid conditions for a more thorough understanding.

Based on the findings, targeted risk minimization strategies are essential to enhance the safety of CsA and TAC in pediatric nephrotic syndrome patients. Regular monitoring of renal function, metabolic parameters, and neurological status should be implemented to enable early detection and intervention for adverse events. Individualized treatment plans, including adjustments to dosages and treatment durations based on patient-specific factors such as age, gender, and comorbidities, are crucial to minimizing risks. Additionally, increasing awareness among healthcare professionals about the potential adverse effects of these drugs and emphasizing adherence to evidence-based treatment guidelines can improve clinical outcomes. Strengthening pharmacovigilance systems to improve the accuracy and completeness of adverse event reporting and updating product labels to reflect emerging safety concerns are also recommended to ensure safer and more effective use of CsA and TAC.

## 5 Conclusion

Both CsA and TAC can induce nephrotoxicity; however, CsA is associated with reduced urine output and elevated serum creatinine, likely related to hemodynamic factors (with a strong signal for hypertension), while TAC primarily induces renal fibrosis. Additionally, CsA may be linked to neurological damage, whereas TAC is associated with diabetic ketoacidosis and dystonia. Notably, TAC is more prone to inappropriate clinical use, such as off-label and unapproved indication use.

## Data Availability

Publicly available datasets were analyzed in this study. This data can be found here: https://openvigil.sourceforge.net.

## References

[1] Eddy AA Symons JM. Nephrotic syndrome in childhood. Lancet. (2003) 362(9384):629–39. 10.1016/S0140-6736(03)14184-0 12944064

[2] Trautmann A Schnaidt S Lipska-Zietkiewicz BS Bodria M Ozaltin F Emma F Long-term outcome of steroid-resistant nephrotic syndrome in children. J Am Soc Nephrol. (2017) 28(10):3055–65. 10.1681/ASN.2016101121 28566477 PMC5619960

[3] Niaudet P Habib R Tete MJ Hinglais N Broyer M. Cyclosporin in the treatment of idiopathic nephrotic syndrome in children. Pediatr Nephrol. (1987) 1(4):566–73. 10.1007/BF00853590 3153333

[4] Hodson EM Wong SC Willis NS Craig JC. Interventions for idiopathic steroid-resistant nephrotic syndrome in children. Cochrane Database Syst Rev. (2016) 10(10):CD003594. 10.1002/14651858.CD003594.pub5 27726125 PMC6457874

[5] Wu Q Wang X Nepovimova E Wang Y Yang H Kuca K. Mechanism of cyclosporine A nephrotoxicity: oxidative stress, autophagy, and signalings. Food Chem Toxicol. (2018) 118:889–907. 10.1016/j.fct.2018.06.054 29960018

[6] Farouk SS Rein JL. The many faces of calcineurin inhibitor toxicity-what the FK? Adv Chronic Kidney Dis. (2020) 27(1):56–66. 10.1053/j.ackd.2019.08.006 32147003 PMC7080294

[7] Wang W Xia Y Mao J Chen Y Wang D Shen H Treatment of tacrolimus or cyclosporine A in children with idiopathic nephrotic syndrome. Pediatr Nephrol. (2012) 27(11):2073–9. 10.1007/s00467-012-2228-3 22714672

[8] Guo HL Xu J Sun JY Li L Guo HL Jing X Tacrolimus treatment in childhood refractory nephrotic syndrome: a retrospective study on efficacy, therapeutic drug monitoring, and contributing factors to variable blood tacrolimus levels. Int Immunopharmacol. (2020) 81:106290. 10.1016/j.intimp.2020.106290 32058933

[9] Sherman RE Anderson SA Dal Pan GJ Gray GW Gross T Hunter NL Real-world evidence—what is it and what can it tell US? N Engl J Med. (2016) 375(23):2293–7. 10.1056/NEJMsb1609216 27959688

[10] Harpaz R DuMouchel W LePendu P Bauer-Mehren A Ryan P Shah NH. Performance of pharmacovigilance signal-detection algorithms for the FDA adverse event reporting system. Clin Pharmacol Ther. (2013) 93(6):539–46. 10.1038/clpt.2013.24 23571771 PMC3857139

[11] Bohm R Hocker J Cascorbi I Herdegen T. Openvigil–free eyeballs on AERS pharmacovigilance data. Nat Biotechnol. (2012) 30(2):137–8. 10.1038/nbt.2113 22318027

[12] Evans SJ Waller PC Davis S. Use of proportional reporting ratios (PRRs) for signal generation from spontaneous adverse drug reaction reports. Pharmacoepidemiol Drug Saf. (2001) 10(6):483–6. 10.1002/pds.677 11828828

[13] Pozsgai K Szucs G Konig-Peter A Balazs O Vajda P Botz L Analysis of pharmacovigilance databases for spontaneous reports of adverse drug reactions related to substandard and falsified medical products: a descriptive study. Front Pharmacol. (2022) 13:964399. 10.3389/fphar.2022.964399 36147337 PMC9485933

[14] Sakaeda T Tamon A Kadoyama K Okuno Y. Data mining of the public version of the FDA adverse event reporting system. Int J Med Sci. (2013) 10(7):796–803. 10.7150/ijms.6048 23794943 PMC3689877

[15] Naesens M Kuypers DR Sarwal M. Calcineurin inhibitor nephrotoxicity. Clin J Am Soc Nephrol. (2009) 4(2):481–508. 10.2215/CJN.04800908 19218475

[16] Yilmaz DE Kirschner K Demirci H Himmerkus N Bachmann S Mutig K. Immunosuppressive calcineurin inhibitor cyclosporine A induces proapoptotic endoplasmic reticulum stress in renal tubular cells. J Biol Chem. (2022) 298(3):101589. 10.1016/j.jbc.2022.101589 35033536 PMC8857494

[17] Textor SC Canzanello VJ Taler SJ Wilson DJ Schwartz LL Augustine JE Cyclosporine-induced hypertension after transplantation. Mayo Clin Proc. (1994) 69(12):1182–93. 10.1016/S0025-6196(12)65772-3 7967781

[18] Scherrer U Vissing SF Morgan BJ Rollins JA Tindall RS Ring S Cyclosporine-induced sympathetic activation and hypertension after heart transplantation. N Engl J Med. (1990) 323(11):693–9. 10.1056/NEJM199009133231101 2388667

[19] Ventura HO Milani RV Lavie CJ Smart FW Stapleton DD Toups TS Cyclosporine-induced hypertension. Efficacy of omega-3 fatty acids in patients after cardiac transplantation. Circulation. (1993) 88(5 Pt 2):II281–5. 8222166

[20] Sudano I Farshad M Flammer AJ Spieker L Periat D Enseleit F. Acute effect of nitroglycerin on cyclosporine-induced hypertension after cardiac transplantation. Swiss Med Wkly. (2010) 140(9–10):139–45. 10.4414/smw.2010.12966 20131116

[21] Louhelainen M Merasto S Finckenberg P Lapatto R Cheng ZJ Mervaala EM. Lipoic acid supplementation prevents cyclosporine-induced hypertension and nephrotoxicity in spontaneously hypertensive rats. J Hypertens. (2006) 24(5):947–56. 10.1097/01.hjh.0000222766.37971.9f 16612258

[22] Huenges K Kolat P Panholzer B Haneya A. CSA-induced PRES after heart transplantation-report of two cases and review. Thorac Cardiovasc Surg Rep. (2021) 10(1):e59–60. 10.1055/s-0041-1732344 34777943 PMC8580731

[23] Coe CL Horst SN Izzy MJ. Neurologic toxicities associated with tumor necrosis factor inhibitors and calcineurin inhibitors. Neurol Clin. (2020) 38(4):937–51. 10.1016/j.ncl.2020.07.009 33040870

[24] Gijtenbeek JM Van Den Bent MJ Vecht CJ. Cyclosporine neurotoxicity: a review. J Neurol. (1999) 246(5):339–46. 10.1007/s004150050360 10399863

[25] Danish A Mughal SI Zaidi U Dildar S Samad S Jamal A Frequency and risk factors of cyclosporine-induced neurotoxicity in allogeneic stem cell transplant recipients. Cureus. (2021) 13(11):e19824. 10.7759/cureus.19824 34963841 PMC8696087

[26] Danish A Mughal SI Zaidi U Dildar S Samad S Jamal A [Posterior reversible encephalopathy syndrome]. Srp Arh Celok Lek. (2003) 131(11–12):461–6. 10.2298/SARH0312461P 15114789

[27] Schwartz RB Bravo SM Klufas RA Hsu L Barnes PD Robson CD Cyclosporine neurotoxicity and its relationship to hypertensive encephalopathy: CT and MR findings in 16 cases. AJR Am J Roentgenol. (1995) 165(3):627–31. 10.2214/ajr.165.3.7645483 7645483

[28] Chen LW Chen JS Tu YF Wang ST Wang LW Tsai YS Age-dependent vulnerability of cyclosporine-associated encephalopathy in children. Eur J Paediatr Neurol. (2015) 19(4):464–71. 10.1016/j.ejpn.2015.02.003 25769225

[29] Ume AC Wenegieme TY Shelby JN Paul-Onyia CDB Waite AMJ 3rd Kamau JK Tacrolimus induces fibroblast-to-myofibroblast transition via a TGF-beta-dependent mechanism to contribute to renal fibrosis. Am J Physiol Renal Physiol. (2023) 324(5):F433–45. 10.1152/ajprenal.00226.2022 36927118 PMC10085566

[30] Liu L Guo J Pang XL Shang WJ Wang ZG Wang JX Exploration of the mechanism of NORAD activation of TGF-beta1/Smad3 through miR-136-5p and promotion of tacrolimus-induced renal fibrosis. Ren Fail. (2023) 45(1):2147083. 10.1080/0886022X.2022.2147083 36748746 PMC9930837

[31] Xiong D Yue B Ye S Wang H Ban L Chen Y The impact of long-term exposure to tacrolimus on chronic kidney disease after lung transplantation: a retrospective analysis from a single transplantation center. Transpl Immunol. (2023) 78:101810. 10.1016/j.trim.2023.101810 36918103

[32] Ozbay LA Smidt K Mortensen DM Carstens J Jorgensen KA Rungby J. Cyclosporin and tacrolimus impair insulin secretion and transcriptional regulation in INS-1E beta-cells. Br J Pharmacol. (2011) 162(1):136–46. 10.1111/j.1476-5381.2010.01018.x 20825407 PMC3012412

[33] Rodriguez-Rodriguez AE Porrini E Torres A. Beta-cell dysfunction induced by tacrolimus: a way to explain type 2 diabetes? Int J Mol Sci. (2021) 22(19):10311. 10.3390/ijms221910311 34638652 PMC8509035

[34] Chakkera HA Kudva Y Kaplan B. Calcineurin inhibitors: pharmacologic mechanisms impacting both insulin resistance and insulin secretion leading to glucose dysregulation and diabetes Mellitus. Clin Pharmacol Ther. (2017) 101(1):114–20. 10.1002/cpt.546 27804122

[35] Trinanes J Rodriguez-Rodriguez AE Brito-Casillas Y Wagner A De Vries APJ Cuesto G Deciphering tacrolimus-induced toxicity in pancreatic beta cells. Am J Transplant. (2017) 17(11):2829–40. 10.1111/ajt.14323 28432716

[36] Lim JH Hwang I Cho JH Kwon E Jung HY Choi JY Impact of conversion from cyclosporine to tacrolimus on glucose metabolism and cardiovascular risk profiles in long-term stable kidney transplant recipients. Transplant Proc. (2019) 51(8):2697–703. 10.1016/j.transproceed.2019.04.081 31439330

[37] Miklavcic P Avcin S Jazbec J Vipotnik Vesnaver T Todorova B Trost M Cyclosporine A induced dystonia-parkinsonism. J Neurol Sci. (2017) 375:68–70. 10.1016/j.jns.2017.01.043 28320190

[38] Taque S Peudenier S Gie S Rambeau M Gandemer V Bridoux L Central neurotoxicity of cyclosporine in two children with nephrotic syndrome. Pediatr Nephrol. (2004) 19(3):276–80. 10.1007/s00467-003-1347-2 14758527

[39] do Val Lima PR Ronconi KS Morra EA Rodrigues PL Ávila RA Merlo E Testosterone deficiency impairs cardiac interfibrillar mitochondrial function and myocardial contractility while inducing oxidative stress. Front Endocrinol (Lausanne). (2023) 14:1206387. 10.3389/fendo.2023.1206387 37780627 PMC10534000

[40] Elzokm SS Fouda MA Abdel Moneim RA El-Mas MM. Distinct effects of calcineurin dependent and independent immunosuppressants on endotoxaemia-induced nephrotoxicity in rats: role of androgens. Clin Exp Pharmacol Physiol. (2021) 48(9):1261–70. 10.1111/1440-1681.13526 34042216

